# Influence of selected cognitive performances on musculoskeletal injury occurrence in adult male professional Slovenian PrvaLiga football players in a prospective cohort study

**DOI:** 10.1038/s41598-025-16643-9

**Published:** 2025-08-22

**Authors:** Florian Giesche, Manca Peskar, Luka Šlosar, Boštjan Šimunič, Rado Pišot, Uros Marusic

**Affiliations:** 1 Institute of Occupational, Social and Environmental Medicine, Division of Preventive and Sports Medicine, Goethe University, Frankfurt, Germany; 2 Science and Research Centre Koper, Institute for Kinesiology Research, Koper, Slovenia; 3 Biological Psychology and Neuroergonomics, Department of Psychology and Ergonomics, Faculty V: Mechanical Engineering and Transport Systems, Technische Universität Berlin, Berlin, Germany; 4 Department of Health Sciences, Alma Mater Europaea University, Maribor, Slovenia

**Keywords:** Neurocognitive testing, Cognitive-motor tests, Sports injury, Risk screening, Cognitive neuroscience, Risk factors

## Abstract

Evidence suggests athletes with lower baseline cognitive performance are at higher risk of musculoskeletal injuries. This prospective cohort study investigates basic and executive cognitive functions in predicting injuries in 78 professional male football (soccer) players from four Slovenian first league teams. Data were collected during the 2018/2019 winter break, and injuries recorded in the second half of that and the 2019/2020 season. Cognitive functions assessed by computerized tests (PsyToolkit) included psychomotor vigilance (simple, choice reaction time) and visuospatial memory (Corsi-block-tapping-test), while pen-and-paper tests assessed motor speed, visual scanning, and executive functions (TMT; Delis-Kaplan-Executive-Function-System). Forty-two players sustained at least one musculoskeletal injury (9 contact injuries), 36 remained injury-free. Logistic regression analyses indicated that none of the cognitive measures significantly influenced injury occurrence (*p* > 0.05). However, non-significant trends were observed for the TMT ratio score (*p* = 0.065, OR = 1.64), reflecting cognitive flexibility, and for TMT-A (*p* = 0.05, OR = 0.60), reflecting visual scanning. Specifically, players with lower cognitive flexibility showed a 64% increase in the odds of sustaining an injury, while better visual scanning performance was associated with a 40% reduction in injury odds. No significant association was found between basic or executive cognitive functions and musculoskeletal injuries in professional male football players. However, a non-significant trend suggested that lower cognitive flexibility may be associated with increased injury risk. These findings underscore the need for larger studies to better clarify the role of executive functions in assessing injury risk in football.

## Introduction

Football (soccer) is one of the sports with the highest incidence of musculoskeletal injuries^1^. The overall injury incidence in professional male football players is approximately 8.1 injuries per 1000 h of exposure, with lower extremity injuries being the most prevalent at 6.8 injuries per 1000 h (about 84%)^2^. Within the lower limbs, the thigh, knee, and ankle are the most frequently affected anatomical regions, with incidence rates ranging rom 1.1 to 1.8 injuries per 1000 h, accounting for about 50% of all injuries^2^. Direct physical contact with an opponent accounts for approximately half of all traumatic injuries, followed by non-contact or indirect contact mechanisms such as landing, cuuting or sprinting, which contribute to about 30% of injuries^3^. While numerous neuromuscular (e.g., relative hamstring recruitment, quadriceps strength) and biomechanical factors (e.g., dynamic knee valgus, peak ground reaction forces) have been identified as risk factors—especially for severe injuries such as anterior cruciate ligament (ACL) tears^4^, which involve minimal to no contact in approximately three-quarters of cases^5^—the role of cognitive performance as a potential injury risk factor remains an emerging area of research^6^.

In open-skill sports like football, athletes often interact in unpredictable and dynamic environments, constantly processing a range of external stimuli, like the positions and movements of opponents, teammates, and the ball. This requires quick, spontaneous motor reactions, often under substantial time pressure^7,8^. Successfully interacting with such multi-tasking and time-critical decision-making demands requires adequate perceptual-cognitive abilities^9^. In fact, initial systematic evidence suggests an association between lower perceptual-cognitive performance (e.g. visuomotor speed, simple and complex reaction time, visual short-term memory, attention and concentration, cognitive flexibility and working memory) and less favorable lower limb biomechanics (joint kinematics and kinetics), which may increase the risk of injury (e.g., ACL tears) in athletic tasks such as jump-landings or run-and-cut movements that incorporate cognitive challenges^6,9^. These tasks often involve a time-constrained decision-making component (e.g., visual cues indicating landing side or cutting direction briefly before reaching the force plate)^6,9,10^. This aligns with previous findings showing that team sport athletes with slower preseason visuomotor reaction times in the upper extremity (e.g., button press in response to visual stimulus on a screen or computerized testing using the D2 Dynavision Assessment and Training System) are at increased risk of injury during the subsequent playing season^11,12^. Specifically, those authors reported that a preseason reaction time cut-off of ≥ 0.545 s was associated with nearly three times higher odds of injury (OR = 2.94), while a threshold of ≥ 0.705 s doubled the injury risk (OR = 2.30), underscoring slower visuomotor reaction speed as a meaningful predictor of injury in team sport athletes. Thus, these players may have greater difficulty maintaining optimal motor control during game situations that require rapid, time-critical decision-making, making them more prone to injury^13^.

However, findings for other cognitive domains—such as visual and verbal memory, motor speed, and simple reaction time—have been inconsistent, showing no clear association with injury occurrence in many cases^6,14^. Executive functions^15^ —such as cognitive flexibility (the ability to shift attention between tasks or adjust to new demands), working memory (holding and manipulating information over short periods), and inhibitory control (suppressing automatic or inappropriate responses)—are higher-order cognitive processes that enable complex, goal-directed behavior in team sports^16,17^. While previous research has shown that executive functions are associated with sport performance and success in youth football^16–20^, few studies have examined whether poorer executive function, as reflected by lower test scores, compromises an athlete’s ability to adapt safely and make rapid decisions during dynamic movements under time pressure^6,9,10^. Such cognitive abilities are especially important in football, where players face unpredictable and fast-changing situations^9,16,17^. Poor cognitive flexibility or working memory, for instance, may delay an athlete’s ability to re-plan actions in response to sudden cues and events, while reduced inhibitory control may impair the suppression of inappropriate or outdated movement plans or actions—potentially leading to compromised biomechanics and increased injury risk^21^. Although some research has linked visuomotor processing speed to injury risk as indicated above, there is limited evidence on executive functions as predictors of injury occurrence^14^. The current study addresses this gap by assessing executive functions using established cognitive performance measures, thereby expanding the current understanding of injury risk factors in football.

Thus, the aim of our study was to investigate the potential influence of selected cognitive performance measures, assessed during the preseason using both established computerized and pen-and-paper tests, on the occurrence of musculoskeletal injuries in professional adult male football players during the subsequent playing season. We hypothesized that, in addition to lower-level cognitive processes such as visual perception, visuomotor vigilance, and visuospatial short-term memory, executive function measures would also be associated with injury risk.

## Methods

### Study design and participants

We adopted a prospective cohort study design to investigate cognitive performance and injury risk in professional football players. All players from the Slovenian First Division (PrvaLiga) were eligible for inclusion. To ensure a representative sample, all available players were invited to participate; therefore, no exclusion criteria were applied, and no a priori sample size calculation was performed.

Cognitive data were collected from 151 players across seven clubs during the 2018/2019 winter break. Injury data were subsequently obtained from four of these clubs, resulting in a final analytical sample of 78 players. The reduction in sample size was due to varying levels of cooperation and data availability across clubs, which may have introduced selection bias. No player dropouts occurred during testing; the main limitation was the incomplete injury data from some clubs.

The study was approved by the National Medical Ethics Committee of the Republic of Slovenia (ID: blinded for review) and conducted according to the ethical standards set by the Declaration of Helsinki (Version Fortaleza, 2013).All participants signed a written informed consent before participation.

### Measurements and outcomes

#### Musculoskeletal injuries

Prospective data on musculoskeletal injuries—including injury mechanism (contact or non-contact), number of injuries, affected body region, and days lost due to injury—were independently recorded by the medical and therapeutic staff of each participating team for every player during the second half of the 2018/2019 season and the entirety of the 2019/2020 season. Injuries were recorded and diagnosed by licensed medical staff using each team’s usual reporting and diagnostic procedures in real-world settings. Although no centralized injury database was used, all teams employed qualified staff trained in sports medicine, which supports the ecological validity of the data. To enhance consistency and comparability across teams, we requested that injury documentation follow the general definitions and data collection procedures outlined in the consensus statement on injury definitions and data collection in football^22^. The data were subsequently provided to the research team in pseudonymised form for analysis. In-game exposure time (i.e., the duration each player was actively involved while the team competed against another team) was obtained from official referee match reports. Additional player-specific information—including age, anthropometric characteristics, and playing position (forward, midfielder, defender, or goalkeeper)—was also collected.

#### Cognitive performance testing

**Fig. 1:**
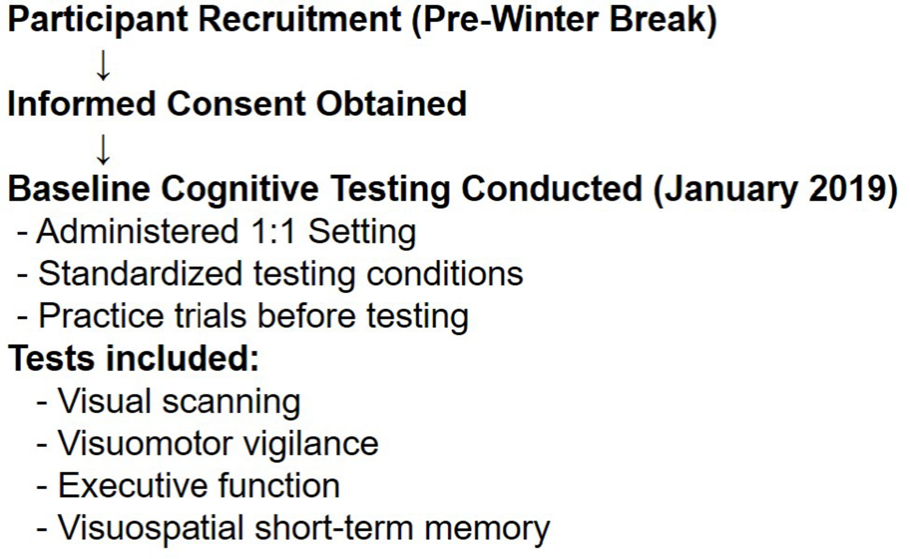
Overview of the cognitive performance data collection procedure conducted during the 2018/2019 winter break.

Baseline performance data from specific cognitive domains—including visual scanning, visuomotor vigilance, executive function, and visuospatial short-term memory—were collected during the winter break of the 2018/2019 season (January 2019). The cognitive assessments were instructed by two experienced coauthors (UM, MP) in a 1:1 setting and under standardized conditions (i.e., instructions, room, temperature, light, chair, and test equipment). Testing was conducted at similar times of day across participants to minimize the effects of diurnal variation in cognitive functioning. All participants practiced each test during a test trial and had sufficient time to ask questions before the actual test started. The following flowchart (Fig. 1) summarizes the data collection process for the cognitive performance testing.

The cognitive assessment comprised the following pen-and-paper and computerized tests: An refined version of the Trail-Making-Test (Delis-Kaplan Executive Function System; TMT)^23^ that consists of five conditions assessing various components of visuomotor sequencing skills was applied. Specifically, TMT-1 assesses visual scanning, TMT-2 number sequencing, TMT-3 letter sequencing, and TMT-5 motor execution speed of the dominant hand (to follow a pre-drawn line). These subtests are generally considered to measure lower-level cognitive process. In contrast, TMT-4 assesses number-letter switching and is considered a measure of executive function, namely cognitive flexibility/set-shifting and working memory^23,24^. The TMT conditions 2 and 4 are conceptually equivalent to the traditional TMT-A and TMT-B^25^. However, the completion times cannot be directly compared due to the different format of the paper sheet (A3 vs. A4). The total time needed to complete each of these conditions was documented. Shorter completion times indicate better cognitive performance and vice versa.

The TMT-2 and TMT-4, as well as the derived TMT ratio score—which expresses the relative increase in completion time from the simpler (TMT-2) to the more complex task (TMT-4), calculated as TMT-4 divided by TMT-2—were used.

The TMT ratio score (TMT-4 divided by TMT-2) was used as the primary outcome for executive functioning. The ratio score reflects performance on the more complex (executive function) task (TMT-4) relative to the simpler baseline task (TMT-2), thereby integrating higher-order processes such as cognitive flexibility and set-shifting with individual processing speed^26^. By adjusting for baseline motor speed and visual scanning, this proportional index offers a nuanced measure of cognitive efficiency—combining basic and complex cognitive processes—and enhances sensitivity to subtle cognitive differences, particularly in high-performing populations like professional football players. Arbuthnott and Frank (2000)^26^ found that the TMT ratio score was more strongly correlated with task-switching performance than the difference score (TMT-4 min TMT-2), suggesting it may be a better indicator of set-shifting ability.

However, to complement the ratio-based approach, the absolute difference score was also reported. Sánchez-Cubillo et al. (2009)^24^ found the difference score to show higher construct validity for isolated executive components, particularly switching and cognitive flexibility. Unlike the ratio, the difference score reflects the absolute additional time required for the more demanding task, without adjusting for baseline processing speed. This suggests that both scores reflect complementary aspects of executive functioning.

While difference scores can offer stronger construct validity for specific executive functions such as cognitive flexibility^24^, they often suffer from low reliability due to the accumulation of shared measurement error between the correlated subcomponents used to compute the derived score^27^. This concern likely applies to the TMT ratio score as well, since it also combines two correlated measures. However, the test–retest reliability of the TMT subtests used in this study is considered high^28^.

Simple and choice reaction times (SRT and CRT, respectively; visuomotor vigilance) were assessed by using the PsyToolkit^29^. In the SRT task, participants were required to respond to a target stimulus (*n* = 20) shown on the computer screen by pressing the spacebar on a standard keyboard as fast as possible with the index finger of their dominant hand. For the CRT task, they needed to respond as quickly as possible to the target stimulus, which was sequentially and in a random order presented in one of four boxes by pressing one of the 4 keys, each corresponding to one box (i.e., left middle finger: “z” key, left index finger: “x” key, right index finger: “,” key, and right middle finger: “.” key).

The Corsi block-tapping test was used in the forward version to assess visuospatial short-term memory^30^. Squares lit up in yellow colour in a random sequence. Participants were asked to repeat the same sequence by marking the corresponding squares with the computer mouse in the same order. The task started with a twosquare sequence and the number increased by one square each trial when the participants responded correctly. When participants conducted an error, the same sequence difficulty (with a different lighting pattern) was presented again. When both consecutive trials were incorrect, the number of the last correct sequence was recorded. A maximum score of 9 could be obtained. A higher score corresponds to better short-term memory performance. This test was subsequently included in the test battery and was therefore not performed by all participants.

### Statistics

Following previous research on cognitive predictors of injury risk^11,12^, we conducted a binary logistic regression to examine the influence of pre-seasonal cognitive performance measures (SRT, CRT, CORSI, TMT-1-5, TMT-ratio-score; independent variables) on the occurrence of musculoskeletal injuries (dependent variable) during the following season in professional football players. These cognitive assessments, among others, have been used in previous research to investigate the relevance of specific cognitive domains—such as visual scanning, visuomotor vigilance, executive function, and visuospatial short-term memory—for predicting injury risk in athletic populations^6,14^.

In addition to cognitive performance measures, age (years) and total game exposure time (minutes) were included as independent variables in the analysis. All characteric variables (e.g. anthropometrics, age, total game exposure time, total number of games, playing positions) were reported as mean and standard deviations. Potential differences between groups were identified using independent t-tests and Chi-square tests (playing positions). Players with no game exposure were assigned a value of 0 min.

Z-scores for each independent variable in the binary logistic regression were calculated by standardizing the variables (subtracting the mean and dividing by the standard deviation) to enable comparability of effect sizes across predictors. Stepwise backward elimination was applied, removing the independent variables with the highest p-values to identify the most influential predictors. Z-values, p-values, and odds ratios (OR) as well as sensitivity and specificity values were reported for both the overall (including all independent variables) and reduced models. Model fit was assessed using AIC, BIC, R² (Nagelkerke and Cox & Snell), and p-values.

The dataset was randomly split into a training set (54 cases; 70%) and a test set (24 cases; 30%) using random number generation for overall model validation, with approximately equal proportions of injured and non-injured players in both sets. Separate regressions were conducted, and the results were compared to assess overfitting, model stability, and the generalizability of the model to new data.

To check for linearity, predicted probabilities were calculated and visually assessed for each independent variable. A log-transformation of the game exposure time variable substantially improved model fit (AIC: 96 to 81; BIC: 109 to 94; R² Nagelkerke: 0.19 to 0.29; R² Cox & Snell: 0.14 to 0.22, p: 0.071 to 0.012), suggesting a non-linear relationship with injury risk. The transformed variable was therefore retained in the main binary logistic regression model including five independent variables (SR, CRT, TMT-ratio-score, age, and game exposure time).

Variables with high multicollinearity (VIF > 3) were excluded from the overall model and examined separately in exploratory analyses. This applied to the TMT-2, TMT-4, and the absolute TMT difference score, which are mathematically related to the TMT ratio score and therefore highly collinear. TMT-1, TMT-3, and TMT-5 were also analyzed in separate models to maintain conceptual consistency and model parsimony, although they did not show problematic collinearity with the TMT ratio score. Results for the CORSI test were missing for the first 25 out of 78 players tested (33%), as the test was introduced later in the assessment battery. Because injury status was unknown at the time of testing, the missing data are likely missing at random (MAR), which reduces the risk of systematic bias. Due to the substantial proportion of missing data, imputation was deemed inappropriate, as it could have introduced distortion into the primary model. Therefore, the CORSI test was excluded from the main analysis and its potential influence was investigated separately in the subset of players for whom data were available. Missing TMT scores (i.e., TMT-2 and − 4) were observed for five players because they were unaware of the correct order of letters in the alphabet. For all other independent variables complete data were available.

Potential outliers were identified using boxplots (values > 1.5 interquartile range) (TMT-ratio-score: 4 outliers, TMT-2: 4 outliers, SRT: 2 outliers, CRT: 1 outlier). Since the plausibility of these values could not be excluded (cognitive test results) and outliers appeared in both groups, all values were retained in the analysis. However, sensitivity analyses were conducted to assess the impact of outliers. Nine out of 42 players sustained injuries due to direct opponent contact. To include as many players as possible while accounting for the potential influence of the injury mechanism, binary logistic regression was performed with and without these players. The significance level was set at *p* < 0.05. All analyses were conducted using Jamovi (Version 2.2.5).

## Results

### Characteristics

In total, 78 players underwent pre-season cognitive testing, of whom 42 (54%) players sustained at least one musculoskeletal injury (33 non-contact and 9 contact injuries), while 36 (46%) players remained injury-free during the observed following playing season. In total, 51 (82%) non-contact (Mean ± SD: 1.6 ± 1.2, range: 1 to 7 injuries) and 10 (18%) contact injuries (1.1 ± 0.3, range: 1 to 2 injuries) occurred. Thirty-one players experienced 1 injury, seven players experienced 2 injuries, three players experienced 3 injuries, and one player experienced 7 injuries, resulting in a total downtime of 34 ± 51 days (Mean ± SD), ranging from 0 to 289 days. Of all recorded injuries, 50 (81%) affected the lower limbs (ankle: 9%, knee: 23%, hip: 7%, pelvis: 5%, achilles tendon: 7%, calf muscles 11%, quadriceps and/or hamstrings: 32%, and abductor or adductor muscles: 6%), while 11 (19%) affected the trunk (e.g. back or rips) or upper limbs (shoulders or hands). Both the injured and uninjured groups tended to differ in terms of age, with the difference approaching statistical significance, but not reaching it. However, with regard to anthropometrics, the distribution of playing positions, total game exposure time, and number of matches during the observed season, no significant differences between the two groups were observed (Table 1). Eight players (7 uninjured players) were not used in any game (non-starters) and therefore had no game exposure. Data of all players were considered for statistical analysis.

**Table 1:** Characteristics of the two groups.

	Injured (*n* = 42)	Uninjured (*n* = 36)	*p*-value
	Mean ± SD	Mean ± SD	
Age (years)	25.2 ± 5	23.3 ± 4	0.057
Height (cm)	183 ± 7	182 ± 6	0.664
Body weight (kg)	78 ± 7	77 ± 6	0.371
BMI (kg/m^2^)	23 ± 1	23 ± 1	0.600
Total game exposure time (min.)	1.962 ± 1.090 (range: 0–4.103)	1.654 ± 1.388 (range: 0–4.681)	0.276
Total number of games (n)	26 ± 12 (range: 0–51)	24 ± 19 (range: 0–56)	0.468
**Playing position**	**Frequency**	**Frequency**	
Goal (n/%)	5 (12%)	8 (22%)	x^2^-Test = 5.4, *p* = 0.144
Defense (n/%)	17 (40%)	12 (33%)	
Midfield (n/%)	18 (43%)	10 (28%)	
Forward (n/%)	2 (5%)	6 (17%)	

Abbreviations: min = minutes, n = numbers, SD = standard deviation.

### Model fit validation and generalizability

For the overall model, including all five independent variables across all players, the training set (54 cases) yielded an AIC of 58, BIC of 68, R² CS of 0.22, R² Nagelkerkes of 0.29, and a p-value of 0.073. The test set (24 cases) resulted in an AIC of 28, BIC of 34, R² CS of 0.44, R² Nagelkerkes of 0.59, and a p-value of 0.040. These findings suggest that the model performs better on the test set, indicating that overfitting to the training data is unlikely. Furthermore, the improved performance on the test set demonstrates that the model generalizes effectively to new, unseen data.

### Main binary logistic regression analysis

The overall model showed good fit (AIC = 81; BIC = 94; Nagelkerke R² = 0.29; Cox & Snell R² = 0.22; *p* = 0.012). Within this model, only the TMT ratio score was significantly associated with injury occurrence (see Table 2). The model achieved a sensitivity of 0.82 and a specificity of 0.56.

**Table 2:** Descriptive statistics of independent variables of the two groups and the results of the initital binary regression model (dependent variable: injured vs. uninjured) based on Z-standardised values.

	Descriptive statistics of independent variables		Binary regression model (injured vs. uninjured)				
	Injured (*n* = 42)	Uninjured (*n* = 36)	B	Z	*p*	95%-CI	OR
SRT	276 ± 28	277 ± 23	-0.81	-1.8	0.078	0.18 to 1.10	0.45
CRT	452 ± 54	441 ± 56	0.52	1.4	0.148	0.83 to 3.41	1.68
TMT-ratio-score	2.5 ± 0.8	2.2 ± 0.5	0.84	2.2	**0.027**	1.10 to 4.85	2.30
Age	25.2 ± 5	23.3 ± 4	0.68	1.9	0.060	0.97 to 3.96	1.96
Total game exposure time (min.)*	1.962 ± 1.090	1.654 ± 1.388	0.37	1.1	0.261	0.76 to 2.71	1.44

Abbreviations: SRT = simple reaction time, CRT = choice reaction time, TMT = Trail-Making-Test, n = numerus (injured/ uninjured), SD = standard deviation, df = degrees of freedom, CI = confidence intervals, *log-transformed.

**Table 3:** Overview about main Stepwise logistic regression model reduction for the main binary logistic analysis based on Z-standardised values.

Step	Variable removed	Beta	Z-Value	*p*-Value	Odds Ratio (OR)	Confidence Interval (CI)
1	Game exposure Time	0.37	1.12	0.261	1.44	0.76 to 2.71
2	CRT	0.31	1.0	0.319	1.36	0.74 to 2.51
3	SRT	-0.34	-1.0	0.302	0.71	0.37 to 1.36
4	Age	0.47	1.8	0.071	1.59	0.96 to 2.63
Final	TMT-Ratio-Score	0.49	1.9	0.065	1.64	0.97 to 2.77

After stepwise removal of variables with the highest p-values, the analysis proceeded as follows: game exposure time was excluded in the first step, followed by CRT in the second step, SRT in the third step, and age in the fourth step. In the reduced model, only the TMT ratio score remained, showing a trend toward significance and suggesting a potential—but statistically non-significant—association with injury occurrence (sensitivity = 0.55, specificity = 0.57; Table 3). Players with poorer cognitive flexibility (i.e., higher TMT ratio scores) showed 64% higher odds of injury, although the 95% confidence interval included 1.

Excluding participants with contact injuries resulted in similar outcomes, except in step four, where the TMT-ratio-score was excluded (B = 0.49, Z = 1.8, *p* = 0.078, OR = 1.64, CI: 0.95 to 2.83), and age became significant (B = 0.55, Z = 2.1, *p* = 0.040, OR = 1.74, CI: 1.03 to 2.95), yielding a sensitivity of 0.42 and specificity of 0.75. The exclusion of outliers did not notably change the results.

### Separate exploratory binary logistic regression analyses

Each exploratory regression model included one cognitive test variable along with age and game exposure time. All variables were z-standardized. Model fit indices for the TMT-based exploratory models showed comparable results, with AIC values ranging from 92 to 96, BIC from 101 to 105, Nagelkerke R² from 0.10 to 0.19, Cox & Snell R² from 0.07 to 0.14, and p from 0.14 to 0.179. The model including the CORSI task showed better AIC and BIC values (AIC = 70; BIC = 77), but its explained variance (Nagelkerke R² = 0.13; Cox & Snell R² = 0.10) fell within the range of the TMT models, and the predictor was not statistically significant either (*p* = 0.166).

For the model including TMT-1, significant effects were found for TMT-1 (*p* = 0.016, OR = 0.49, 95%-CI: 0.27 to 0.87) and age (*p* = 0.014, OR = 2.3, 95%-CI: 1.18 to 4.49), but not game exposure time (*p* = 0.775, OR = 0.93, 95%-CI: 0.55 to 1.55). After stepwise removal of variables, game exposure time was excluded first, followed by age (*p* = 0.019, OR = 2.00, CI: 1.12 to 3.59). In the reduced model, TMT-1 showed a trend toward significance (*p* = 0.050, OR = 0.60, 95% CI: 0.36 to 1.00), suggesting that better visual perception reduced the odds of injury by approximately 40%. Similar results were found for TMT-5 (*p* = 0.068, OR = 0.64, 95% CI: 0.39 to 1.03), indicating that faster motor execution speed may reduce injury risk by around 36%. However, in both cases, the 95% confidence intervals included 1, and thus the effects should be interpreted with caution. No significant effects were found for TMT-2 (*p* = 0.416, OR = 0.82, 95%-CI: 0.52 to 1.31), TMT-3 (*p* = 0.162, OR = 0.67, 95%-CI: 0.38 to 1.18), TMT-4 (*p* = 0.709, OR = 1.09, 95%-CI: 0.69 to 1.74), absolute TMT difference score (*p* = 0.450, OR = 1.20, 95%-CI: 0.74 to 1.95), or CORSI (*p* = 0.951, OR = 0.98, 95%-CI: 0.57 to 1.69). The exclusion of outliers did not notably change the results.

## Discussion

Our data suggest a possible but non-significant association between individual basic and complex (executive) cognitive functions and muscoloskelettal injuries in professional male football players. However, due to the non-significant results across the assessed cognitive functions, our hypothesis could not be confirmed.

Although not statistically significant, we found that players with higher TMT-ratio-scores—indicating poorer cognitive flexibility or set-shifting ability—had 64% higher odds of injury. Athletes in open-skill sports like football must quickly adapt to unpredictable changes on the field (e.g., positions of teammates, opponents, and the ball) while making rapid decisions (e.g., pass, tackle, dribble) under time constraints^16^. This constant adjustment may require cognitive flexibility^31^. Athletes with lower cognitive flexibility may struggle to react and adapt motor responses to unexpected events, increasing their injury risk. Initial evidence suggests that lower cognitive performance, such as reduced cognitive flexibility, may lead to unfavourable biomechanics during jump landing tasks, which involve time-sensitive decisions and may increase the risk of knee injuries^21^. In this context, the observed potential association between higher TMT ratio scores and increased injury risk in the present study appears plausible.

Interestingly, performance on the TMT-4 test version, as well as the magnitude of the absolute TMT difference score—both measures of executive functions such as working memory and cognitive flexibility^24^—were far from sigmificantly related to injury risk. This may suggest that the proportional index of the TMT-ratio-score, which expresses executive performance relative to basic processing abilities, provides a more nuanced and sensitive measure of cognitive efficiency related to injury risk in professional football players. This makes sense insofar as playing performance and potential biomechanical injury risk factors in motor-cognitively challenging game situations seem to depend on a combination of basic and complex cognitive processes^6,7,9,16^, rather than a more isolated, specific domain like working memory or cognitive flexibility, which are predominantly reflected by the TMT-4 and the absolute difference score^24^. It is important to emphasize that this finding is hypothesis-generating and requires further investigation in larger, adequately powered studies. However, since the predictive value of the TMT-ratio-score was not statistically significant, showed only moderate sensitivity and specificity, and was only marginally better than age as a predictor—combined with its lower test-retest reliability compared to the individual TMT subtests^28^—this trend should be interpreted with caution. Future prospective studies should further explore and confirm the injury-predictive potential of these cognitive functions using the TMT or other established tests (e.g., task switching paradigms like PsyToolkit) before drawing more definitive conclusions about the predictive value of cognitive performance measures. Additionally, future research should investigate other executive functions not explicitly assessed in the present study such as working memory and inhibitory control, which are also critical for rapid decision-making during athletic tasks^21^. These domains can improve with training^32^ and may influence injury risk, making them important targets for assessment and prevention.

The association between higher age or more years of playing and an increased risk of injury in football players is well-documented^33,34^ and was also observed in our dataset. This relationship may reflect accumulated musculoskeletal load and degeneration, slower recovery times, or age-related declines in neuromuscular function^35^. Our findings suggest that any predictive influence of cognitive performance should be interpreted within the context of age-related risk. Future studies should further investigate whether cognitive factors interact with or mediate the effects of age on musculoskeletal injury risk.

Regarding more basic cognitive processes such as visual search and perception, we found a potential link to injury risk. Specifically, players with shorter TMT-1 completion times, indicating faster visual search, tended to show approximately 40% decreased odds of injury. However, this effect was not statistically significant, and thus the potential association should be interpreted with caution. Larger, adequately powered confirmatory studies are warranted to explore this relationship further. Previous studies have suggested that injured athletes might outperform uninjured ones on visual perception tests such as the TMT-1. For example, Stone et al.^36^ proposed that athletes recovering from ACL injuries might adopt a postoperative strategy that compensates for proprioceptive deficits by increasing visual control. This cortical compensatory mechanism has been supported by previous systematic evidence^37^. However, since injury history (e.g., prior ACL injuries) was not explicitly assessed in our sample, we cannot determine whether such a mechanism was present among the injured players in this study. As a result, any interpretation regarding visual compensation influencing TMT performance remains speculative and should be treated with caution. The same applies to the observed, non-significant trend between faster TMT-5 performance—indicative of faster hand motor execution speed—and an approximately 36% reduction in injury odds. Future research should explore whether pure motor speed represents an additional injury risk factor in football players, beyond cognitive processing speed.

Contrary to our hypothesis, we did not find a relationship between injury risk and either visuomotor vigilance (i.e., simple and choice reaction speed) or visuospatial short-term memory (CORSI). Regarding visuomotor vigilance, our findings diverge from previous evidence. Systematic reviews have reported that 7 out of 9^14^ and 5 out of 6 studies^6^ identified a link between slower upper-extremity visuomotor speed and increased injury risk, primarily in team sport athletes. Several methodological and participant-related differences may account for this discrepancy. First, the methods used to assess visuomotor reaction time varied. For example, one study^12^ included in the reviews used the Dynavision Assessment and Training System, which involves standing participants completing reaction time tasks with upper extremity movements combined with visual search. This setup offers higher ecological validity, as it more closely mimics the visuomotor demands of real sports. In contrast, our test involved seated participants responding to visual stimuli on a computer screen via key presses, which requires lower motor demands and may therefore underestimate real-world cognitive–motor challenges. Although some of the other reviewed studies also used PC-based reaction tests in a seated position, most employed the ImPACT neurocognitive test battery, which may differ slightly from the tasks used in our PsyToolkit-based assessments. Second, participant characteristics differed substantially. Prior research primarily involved high school and collegiate athletes in team sports^6,14^, whereas our sample comprised older, professional male football players. It is possible that those athletes exhibit less variability in basic visuomotor reaction times due to their highly advanced performance levels. However, executive functions—such as cognitive flexibility—may still show meaningful individual differences even at high performance levels, potentially explaining their greater relevance in our findings.

Regarding visuospatial memory, previous reviews reported mixed findings. Four out of 9 studies^14^ and 4 out of 6 studies^6^ examined visual memory (mostly using ImPACT), but only one study (Swanik et al., 2007) in both reviews found a significant relationship with injury risk. Notably, none of these studies used the CORSI test to assess visuospatial memory specifically, limiting direct comparisons. Future research is warranted to determine whether visuospatial memory, as measured by tasks like CORSI, lacks predictive value for injury risk.

### Practical implications and future research

Based on our study’s results, we cannot recommend integrating cognitive tests into pre-season performance and injury risk screenings for injury prevention in professional men’s football. However, beyond basic cognive processes such as visul perception, our findings provide initial indications of the potential relevance of executive function, specifically cognitive flexibility and set-shifting abilities. Further studies are needed to better understand the prognostic value of executive cognitive function measures before making specific recommendations for injury risk screening. Specifically, prospective studies, including female players and other key predictors such as age and years of playing as well as previous or preseason injuries, are needed to better understand the potential additional prognostic value of basic cognitive and particularly executive function measures. With regard to the outcome variable, we also suggest a more detailed analysis of injury occurrence, including mechanisms (contact vs. non-contact), locations (e.g., lower limbs), severity (e.g., downtimes), number and types of injuries (e.g., ligament sprains or tears), which was not feasible due to the limited sample size.

The ecological validity of many cognitive assessments—including those used in the present study, which often involve simple button presses performed in a seated position—has been questioned^38–42^. Such static tests may not adequately reflect the dynamic cognitive–motor demands athletes face in competitive settings, thereby limiting their potential to transfer to real-world performance contexts. Rather than relying on isolated cognitive screenings, practitioners should embed cognitive demands within sport-specific tasks that more accurately reflect real-game conditions. Araujo et al.^39,40^ emphasized the importance of ecological cognition, highlighting the dynamic interaction between perception, decision-making, and action. Although initial evidence suggests that combining cognitive and motor tasks may enhance transfer to on-field performance^43^, more ecologically valid approaches are needed. Agility tasks—such as reactive change-of-direction drills, or unplanned landing and cutting tasks that require time-constrained motor reactions to visual cues, as well as dual-task scenarios (e.g., jumping or cutting combined with simultaneous cognitive demands like counting backward or performing a Stroop interference test)^44^—inherently integrate perceptual, cognitive, and physical demands (e.g. change of direction and velocity^45^.

Agility-based training and assessment tasks offer a promising approach by simultaneously targeting concentric and eccentric strength as well as perceptual and cognitive skills^46^. Both agility and lower extremity strength directly—which are highly related and directly affect acceleration and deceleration cycles in athletes—are critical for team sport performance and may also play a role in injury prevention^47^. By requiring athletes to quickly perceive visual cues, make rapid decisions, and execute explosive movements, such tasks closely replicate the multifactorial demands of actual game situations. This integrated approach reflects sport-specific challenges more accurately than static, isolated tests. Initial evidence supports the effectiveness of agility-based training in improving sprint speed, change-of-direction ability, reaction time, lower-limb strength, and flexibility in team sport athletes^48^. Thus, beyond further exploring the injury predictive value of executive functions, research is needed to determine whether more ecologically valid approaches provide added value for injury risk assessment.

### Limitations

One major advantage of our study is its prospective design, which allows us to quantify the predictive role of various cognitive performance measures on future injury occurrence in professional football players. Another strength is the high homogeneity of the sample, with both groups being similar in key characteristics such as sport, performance level, playing time, and anthropometrics. However, this study is not without limitations: Due to multicollinearity among the various TMT measures, separate regression analyses were necessary, precluding the identification of the single most predictive cognitive outcome within one comprehensive model. Nevertheless, the primary model yielded lower AIC and BIC values, indicating superior parsimony and thereby supporting the chosen analytical approach. Due to the limited sample size, the potential impact of playing positions on cognitive performance was not explored.

Injury diagnoses were made by each team’s medical staff using their usual procedures in real-world settings, which supports the ecological validity of the data, but these diagnoses were not centrally reviewed or validated by the authors. Although all teams employed licensed professionals and followed standardized definitions^22^, some variability in diagnostic accuracy and reporting consistency cannot be ruled out. Only musculoskeletal injuries from the observed seasons were reported, leaving the potential impact of head injuries (e.g., concussions) on cognitive performance and injury risk unclear^49^. Severe primary injuries (e.g., ACL tears) are a significant risk factor for re-injuries^50^, but since previous injuries were not reported, their influence on injury occurrence remains speculative. We only received the total number of injuries and downtime, so it was unclear how downtime was distributed to players with multiple injuries. This limited our ability to examine the effect of cognitive performance on injury severity. Finally, only four teams provided injury data, meaning the exclusion of potential selection bias and the representativeness of the results for the entire league cannot be guaranteed. Furthermore, our results regarding the relationship between cognitive performance and injury risk may only be partially generalizable to players in other national leagues, as varying performance levels could influence this association. However, when considering injury incidence rates alone, our data appear comparable to those reported in other professional leagues. Lopez-Valenciano et al.^2^ demonstrated that injury incidence in the top five European leagues does not significantly differ from that in other countries’ professional leagues. Additionally, we acknowledge limitations including the lack of data on injury type, mechanism, and history (e.g., ACL reconstruction), which could act as confounding variables influencing results and should be considered in future research.

A post-hoc sensitivity analysis based on effect sizes from comparable studies (e.g., Swanik et al., 2007^51^ suggests that a sample of approximately 128 participants would be required to reliably detect medium effects (Cohen’s d = ~ 0.5, Alpha = 0.05, power = 0.80). Our sample of 78 players may therefore have limited statistical power to detect such effects.

## Conclusion

Our data suggest a possible but non-significant association between both basic and executive cognitive functions and musculoskeletal injuries in professional male football players. Although these associations were not statistically significant, initial trends point to a potential relevance of executive functions—particularly cognitive flexibility and set-shifting ability—for injury risk. Due to predominantly null findings and non-significant trends, cognitive testing cannot yet be recommended for pre-season injury risk screening in professional football. Further adequately powered prospective studies are needed to clarify the prognostic value of these cognitive measures. Future research should also explore whether incorporating sport-specific cognitive demands—such as visuomotor reaction speed and executive tasks relevant to gameplay—into motor assessments, such as agility, can enhance injury prediction by increasing ecological validity.

## Data Availability

All the information supporting the findings of this study can be found within the paper. The datasets used and/or analysed during the current study are available from the corresponding author on reasonable request.
